# Evaluating UV exposure and skin cancer prevention behaviours in Canada: a national population-based cross-sectional study

**DOI:** 10.1136/bmjph-2024-001983

**Published:** 2025-04-23

**Authors:** Amina Moustaqim-Barrette, Hibo Rijal, Santina Conte, Mahan Maazi, Johnny Hanna, Alexandra Sarah Victoria Kelly, Alicia Belaiche, Alyson McKenna, Sandra Pelaez, François Lagacé, Ivan V Litvinov

**Affiliations:** 1 McGill University, Montreal, Quebec, Canada; 2 Queen’s University, Kingston, Ontario, Canada; 3 The University of British Columbia, Vancouver, British Columbia, Canada; 4 Laval University, Quebec City, Quebec, Canada; 5 Research Institute of the McGill University Health Centre, Montreal, Quebec, Canada; 6 Faculty of Science, University of Ottawa, Ottawa, Ontario, Canada; 7 University of Montreal, Montreal, Quebec, Canada

**Keywords:** preventive medicine, humans, public health, primary prevention

## Abstract

**Introduction:**

Cutaneous melanoma is a common cancer with increasing incidence and significant economic burden. Sun-protective behaviours are crucial in addressing UV-related skin cancer risk and are responsive to public health intervention. This study provides a comprehensive overview of recent prevalence and trends in sun protection and UV exposure in Canada.

**Methods:**

Using data from the Canadian Community Health Survey (CCHS), we examined sun-protective behaviours and UV exposure in a sample of over 77 000 individuals aged ≥18 years during 2011–2018. The analysis employed multivariable logistic regression, considering factors including age, sex, income and immigration status, with results weighted to reflect the Canadian population. We further evaluated temporal trends in UV exposure and sun-protective practices from 2007 to 2018.

**Results:**

Age, sex, income and immigration status significantly influenced sun-protective behaviours. One-third (33.3%) of respondents reported having had a sunburn in the past 12 months, and most reported irregular or ‘never use’ of sunscreen on their body (64.3%) and face (58.1%). Women had significantly higher odds of using sunscreen on their body and face compared with men (OR 2.85, 95% CI 2.68 to 3.03 and OR 4.22, 95% CI 3.96 to 4.49, respectively). Individuals in the highest income quintile were similarly more likely to use sunscreen on their body and face than those in the lowest income quintile (OR 1.78, 95% CI 1.55 to 2.04 and OR 2.45, 95% CI 2.10 to 2.86, respectively). Temporal trends demonstrated an increasing prevalence of spending 2 hours or more in the sun and a decreasing trend in the use of any sunscreen on the body and face.

**Conclusions:**

The findings highlight disparities in sun protection linked to demographic factors. Public health strategies should target high-risk groups to enhance sun-protective behaviours and reduce melanoma incidence. Future interventions must address these disparities to improve skin cancer prevention.

**WHAT IS ALREADY KNOWN ON THIS TOPIC**

- The rise in cutaneous melanoma cases in Canada presents an important public health challenge.
- Increased incidence may suggest that more individuals are being exposed to sun/UV radiation without adequate preventive measures in place.
- Notably, there have been limited public health initiatives, legislation or guidelines from Canadian federal and provincial governments to address this growing crisis.
- Earlier, we defined cutaneous melanoma (CM) incidence by postal code in Canada and correlated geographic/environmental variables with CM incidence to establish a model explaining how the local environment (UV radiation index, vegetation index, rainfall, heat events, etc) influences CM risk.
- This study adds another layer of understanding of how behaviour contributes to CM individual risk in the country.

**WHAT THIS STUDY ADDS**

- Our study fills critical gaps by providing a national overview of the prevalence of several sun-protective behaviours and UV exposure outcomes across diverse demographic groups in Canada.
- By analysing data from over 77 000 participants, we identified key disparities linked to age, sex, income and other sociodemographic characteristics.
- In addition, we provide the first national assessment of temporal trends in key UV exposure and sun-protective behaviours.
- This comprehensive assessment paints a worrisome picture, offers new insights into the populations most at risk and underscores the need for targeted interventions to enhance skin cancer prevention strategies.

**HOW THIS STUDY MIGHT AFFECT RESEARCH, PRACTICE OR POLICY**

- The combined evidence suggests a pressing need for public health initiatives tailored to address identified disparities in sun protection practices.
- We found significant differences in UV exposure and sun-protective practices across sociodemographic groups, as well as a trend in decreased sunscreen use over time.
- Policymakers and health professionals should focus on increasing awareness and accessibility of sun protection measures among high-risk groups.
- Future research should explore the effectiveness of targeted interventions and develop strategies to reduce melanoma incidence, ultimately contributing to improved public health outcomes.
- Education programmes should be introduced in schools and communities to teach the importance of thorough sun protection, combining sunscreen use with physical barriers such as clothing and shade-seeking behaviour/sun avoidance for Caucasians and for skin of colour and Indigenous individuals.
- These programmes should emphasise that sun protection is a family and community responsibility.

## Introduction

 Cutaneous melanoma (CM) is the eighth most common cancer in Canada, and the age-standardised incidence of CM continues to increase over time. Over 11 000 incident CM cases in Canada^1^ and over 100 000 cases in the USA are projected in 2024. The economic burden of skin cancer alone is projected to be almost US$1 billion in Canada^2^ and >US$20 billion in the USA.^3^

The association between sun-protective behaviours and reduction of UV damage is well-documented, where increasing sun-protective behaviours significantly reduce the risk of both non-melanoma and melanoma skin cancers.^4^,^6^ It is estimated that up to 90% of melanomas occur due to excess UV exposure,^7 8^ making it one of the few cancers with a single causal factor that is directly responsive to population-level primary prevention strategies. In the USA, census data have shown differences in sun-protective behaviours based on age, sex and education level,^9^ and other studies have shown race-related and migrant-related differences,^10^ as well as differences by sexual orientation.^11^ These data suggest that social and socioeconomic factors may impact the prevalence of sun-protective behaviours within certain populations.

We have earlier defined CM incidence and mortality by postal code in Canada^12^,^15^ and correlated geographic/environmental variables with CM incidence to establish a model explaining how local environment (UV radiation index, vegetation index, rainfall, heat events, etc) influences CM risk.^16 17^ We conducted a number of large cross-sectional surveys^11 18 19^ and qualitative focus group^20^ studies to elucidate sun protection behaviours in different parts of the country. The objective of the current study is to provide a comprehensive analysis of key sun-protective behaviour and UV exposure outcomes in the general population. Using data from >77 000 in-person Canadian Community Health Survey (CCHS) interviews conducted between 2011 and 2018, we aimed to understand the prevalence of UV exposure and sun-protective behaviours across different sociodemographic strata of Canadian society. In addition, we assessed temporal trends in sun exposure and protective behaviour outcomes from 2007 to 2018. The weighted sample represents >21 million Canadians and will help inform public health policy and interventions related to targeted sun protection and skin cancer prevention efforts in North America and beyond.

## Materials and methods

### Study design

Data for this study were extracted from the 2007–2018 cycles of the CCHS,^21^ made available through Statistics Canada. The CCHS is a nationally representative cross-sectional survey that collects self-reported data on a variety of health status, health determinants and health service utilisation factors of Canadians aged ≥12 years. In 2005, the CCHS introduced an optional component on sun safety, which provinces could opt into. Participating provinces between 2007 and 2018 included Prince Edward Island, Nova Scotia, New Brunswick, Manitoba, Saskatchewan, Ontario and Quebec. From 2011 to 2018, the CCHS response rates ranged from a low of 59.5% (2015–2016) to a high of 87.4% (2013–2014).^22^

The analysis was limited to individuals aged ≥18 years living in the provinces who opted into the CCHS’ optional sun safety module and were not living with cancer at the time of interview, to capture average-risk individuals. Due to the influence of caregivers and possibly unique determinants of sun-protective behaviours among children and youth, this demographic analysis was deemed beyond the scope of this study. This study used complete case analysis applied to variables of interest. Data from 2011 to 2018 were used in the primary analysis, and data from 2007 to 2018 in the trend analysis.

### Study variables and analyses

We explored several outcome variables related to UV exposure and sun safety behaviours. These include time spent in the sun between 10:00 and 16:00 hours on weekends during summer months (<2 hours v ≥2 hours), incidence of sunburn in the preceding 12 months (yes/no), use of tanning beds/booths in the previous 12 months (yes/no), use of any sunscreen on the body and face (always/often vs sometimes/rarely/never), sun protection factor (SPF) on the body and face (<30 vs SPF ≥30), wearing a hat in the sun (always/often vs sometimes/rarely/never), wearing long pants or skirts in the sun (always/often vs sometimes/rarely/never) and wearing sunglasses in the sun (always/often vs sometimes/rarely/never).

All analyses were conducted using R V.4.3.3,^23^ and statistics were weighted using sampling weights provided by Statistics Canada to provide more accurate estimates of variance and to account for uneven selection probabilities. Data from 2011 to 2018 were used for the primary analyses to ensure representation of the most current practices. We used data from 2011 to 2018 for the primary analysis to provide the latest understanding of sun exposure and protection prevalence. In addition, sunglass use and tanning bed use variables were added into the 2011 and later iterations of the CCHS survey. Thus, these two variables do not appear in the trend subanalysis.

First, frequency distributions were used to describe the overall characteristics of the study. A multivariable logistic regression model was then used to investigate the relationship between several independent variables and several UV exposure and sun protection behaviour outcomes, while controlling for the impact of age, sex and race wherever appropriate. In the CCHS, sex refers to a person’s biological sex, that is, their sex assigned at birth. In this study, the categorisation of race is described by Statistics Canada, which bases its definition of visible minority on the Employment Equity Act (EEA). The EEA describes visible minorities as ‘persons, other than Aboriginal peoples, who are non-Caucasian in race or non-white in colour’. Statistics Canada states that in Canada, this group mainly consists of South Asian, Chinese, Black, Filipino, Arab, Latin American, Southeast Asian, West Asian, Korean and Japanese individuals.^21^ All variables are described on the Statistics Canada website.^21^

Adjusted ORs, 95% CI and p values are reported and visualised using forest plots. Bar charts were used to present trends in each outcome over time, from 2007 to 2018, for both men and women. Bar charts were fit with linear trend lines and p values are reported.

### Patient and public involvement

Patients or the public were not involved in the design, or conduct, or reporting, or dissemination plans of our research.

## Results

### Descriptive Statistics

This study analysed open-source data from the CCHS available through Statistics Canada/Canadian Institute for Health Information (CIHI). Table 1 presents the baseline characteristics of the CCHS study sample (2011–2018). Of the combined 83 806 respondents to the sun safety module between 2011 and 2018, 77 209 (92.1%) were retained after exclusions, representing 21 740 124 Canadians after applying sampling weights. All proportions reported in the text are weighted. The sample was fairly evenly distributed across age groups and between sexes. The majority of individuals (80.9%) resided in Canada’s two most populous provinces, Ontario (50.5%) and Quebec (30.4%), as expected. Most individuals (64.0%) had postsecondary education and were married or living in common-law relationships (61.8%). A total of 3034 respondents (N_weighted_=658 923, 3.2%) were Indigenous, and 25.9% of the sample represented naturalised citizens (immigrants). Income quintiles were distributed as expected.

**Table 1:** Summary characteristics of Canadian Community Health Survey respondents (2011–2018)

Characteristics	Unweighted sampleN_unweighted_=77 209	Weighted sampleN_weighted_=21 740 124
	N (%)	N (%)
Sex		
Male	35 221 (45.6%)	10 658 068 (49.0%)
Female	41 988 (54.4%)	11 082 055 (51.0%)
Age (years)		
18–29	10 622 (13.8%)	4 225 095 (19.4%)
30–39	11 016 (14.3%)	3 658 180 (16.8%)
40–49	10 483 (13.6%)	3 711 919 (17.1%)
50–59	14 010 (18.1%)	3 980 936 (18.3%)
60–69	15 232 (19.7%)	3 378 259 (15.5%)
70+	15 846 (20.5%)	2 785 735 (12.8%)
Race		
White	64 346 (89.5%)	15 830 752 (78.5%)
Visible minority	7536 (10.5%)	4 328 378 (21.5%)
Educational attainment		
Less than secondary school graduation	13 535 (17.8%)	2 796 025 (13.1%)
Secondary school graduation	17 591 (23.1%)	4 894 815 (22.9%)
Postsecondary school	44 885 (59.1%)	13 699 443 (64.0%)
Household income quintile		
1	15 951 (20.7%)	4 249 750 (19.6%)
2	15 708 (20.4%)	4 343 698 (20.0%)
3	15 072 (19.5%)	4 334 873 (19.9%)
4	14 926 (19.4%)	4 381 781 (20.2%)
5	15 476 (20.1%)	4 418 679 (20.3%)
Province		
Manitoba (MB)	10 013 (13.0%)	1 921 620 (8.8%)
New Brunswick (NB)	4454 (5.8%)	596 314 (2.7%)
Ontario (ON)	30 308 (39.3%)	10 983 554 (50.5%)
Quebec (QC)	21 402 (27.7%)	6 611 096 (30.4%)
Saskatchewan (SK)	11 032 (14.3%)	1 627 540 (7.5%)
Immigration status		
Native-born	63 879 (85.4%)	15 446 827 (74.1%)
Naturalised	10 904 (14.6%)	5 399 909 (25.9%)
Indigeneity		
General population/non-Indigenous	62 913 (95.4%)	19 685 206 (96.8%)
Indigenous	3034 (4.6%)	658 923 (3.2%)
Time spent in sun		
0 to <30 min	18 372 (25.2%)	5 075 227 (24.5%)
30 min to <2 hours	21 005 (28.8%)	6 151 264 (29.7%)
2 to <4 hours	20 484 (28.1%)	5 814 710 (28.1%)
4 to 6 hours	13 138 (18.0%)	3 636 973 (17.6%)
Sunburn in the last 12 months		
No	50 931 (67.6%)	14 070 505 (66.7%)
Yes	24 369 (32.4%)	7 030 102 (33.3%)
Tanning bed or booth in the last 12 months		
No	61 902 (96.3%)	18 998 933 (96.4%)
Yes	2356 (3.7%)	713 526 (3.6%)
Uses sunscreen on body		
Sometimes/Rarely/Never	36 537 (65.5%)	10 140 552 (64.3%)
Always/Often	19 273 (34.5%)	5 621 029 (35.7%)
Uses sunscreen on face		
Sometimes/Rarely/Never	32 770 (58.7%)	9 162 107 (58.1%)
Always/Often	23 092 (41.3%)	6 607 368 (41.9%)
SPF of sunscreen used (body)		
≤SPF 29	4977 (17.8%)	1 402 880 (17.1%)
≥SPF 30	22 955 (82.2%)	6 784 965 (82.9%)
SPF of sunscreen used (face)		
≤SPF 29	6249 (20.5%)	1 697 920 (19.2%)
≥SPF 30	24 231 (79.5%)	7 153 015 (80.8%)
Wears hat		
Sometimes/Rarely/Never	34 087 (61.0%)	10 180 339 (64.6%)
Always/Often	21 756 (39.0%)	5 584 157 (35.4%)
Wears long pants/skirt		
Sometimes/Rarely/Never	37 615 (67.4%)	11 114 170 (70.6%)
Always/Often	18 167 (32.6%)	4 635 522 (29.4%)
Wears sunglasses		
Sometimes/Rarely/Never	16 242 (34.1%)	5 184 069 (35.3%)
Always/Often	31 405 (65.9%)	9 485 296 (64.7%)

SPF, sun protection factor.

Most individuals (75.5%) spent at least 30 min in the sun between 10:00 and 16:00 hours on days off during the summer, with 45.7% spending ≥2 hours. A third (33.3%) reported sunburn in the previous 12 months, while 3.6% reported using a tanning bed/booth in the same period. Two-thirds of respondents (64.3%) reported using sunscreen sometimes, rarely or never on the body, while 58.1% of respondents used sunscreen sometimes, rarely or never on the face. Of those that did use sunscreen, the majority used SPF ≥30 on the body (82.9%) and on the face (80.8%). More than two-thirds reported wearing a hat (64.6%) or long pants/skirts (70.6%) sometimes/rarely/never, while most respondents did wear sunglasses often or always (64.7%). Baseline characteristics for the sample used in trend analysis (CCHS sample 2007–2018) are detailed in online supplemental table 1.

### UV exposure and sun-protective behaviours by age group

**Figure 1:**
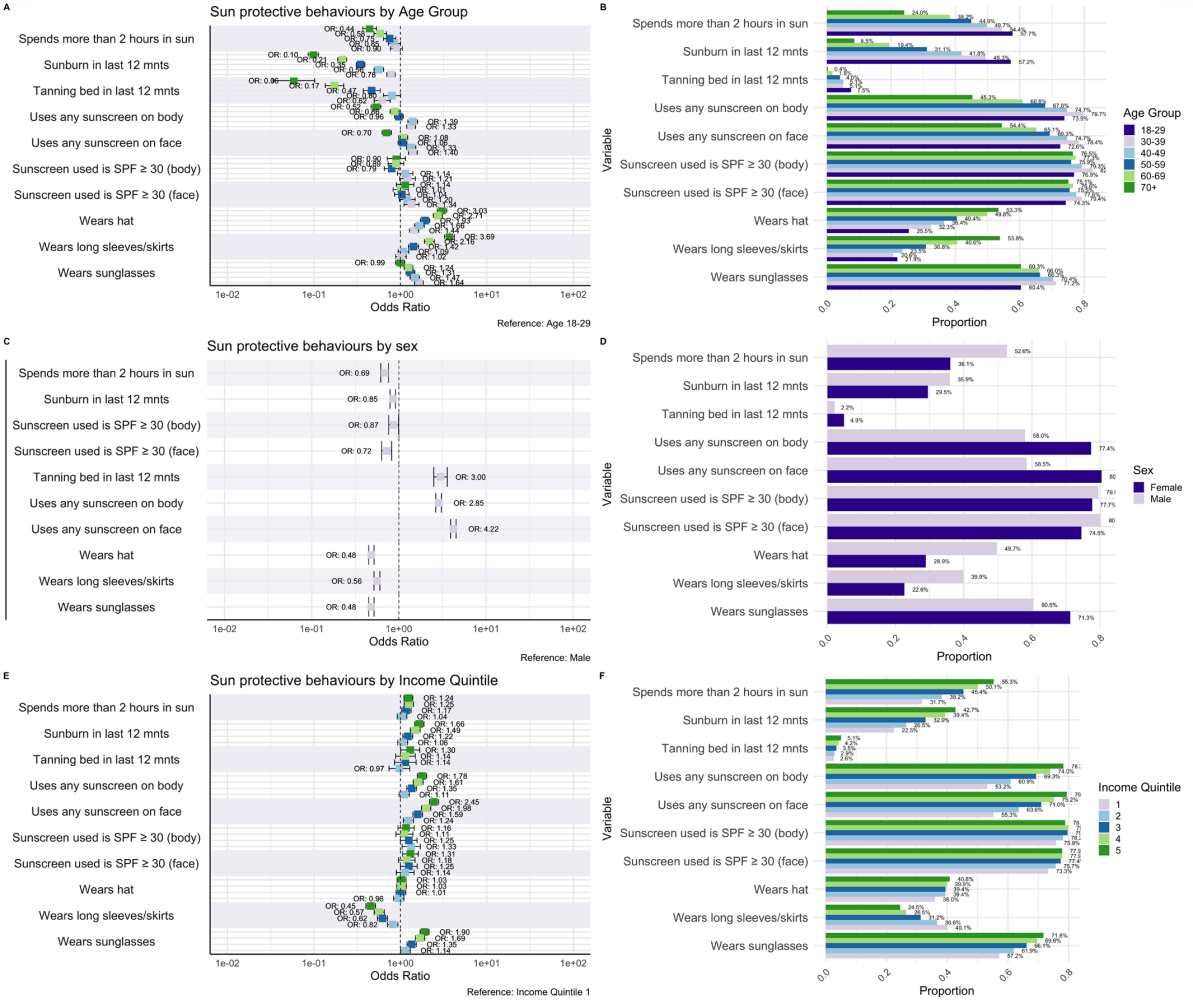
Sun exposure and sun-protective behaviours by age, sex and income. Forest plot comparing sun exposure and protective behaviour outcome variables by age group (**A**), sex (**C**) and income (**E**). Adjusted ORs, controlling for the effect of age, sex and race, where applicable, are presented. Corresponding bar plots comparing the same sun exposure and protective behaviour variables by age group (**B**), sex (**D**) and income (**F**). SPF, sun protection factor.

Our analysis revealed significant age-related differences in UV exposure and sun protection (figure 1A,B). Time spent in the sun was inversely proportional to age. Almost 60% of younger individuals aged 18–29 years reported spending ≥2 hours in the sun, compared with 26.9% of those aged ≥70 years. Incidence of reported sunburn in the past 12 months similarly decreased with age, with the individuals ≥70 years having 90% lower odds of sunburn compared with those aged 18–29 years (OR 0.10, 95% CI 0.06 to 0.11). Tanning bed use was also significantly higher in the youngest age group compared with those aged ≥70 years (OR 0.06, 95% CI 0.03 to 0.10). The use of any sunscreen on the body varied by age, with individuals aged 30–59 years being significantly more likely to use sunscreen on the face and body compared with those aged 18–29 years. Older individuals were significantly less likely to use sunscreen on the face (OR 0.70, 95% CI 0.61 to 0.79) and body (OR 0.52, 95% CI 0.46 to 0.59), compared with those aged 18–29 years. The use of protective clothing increased with age, with those in the ≥70 years age group having 269% higher odds of wearing long pants/skirts (OR 3.69, 95% CI 3.23 to 4.21) and 203% higher odds of wearing hats (OR 3.03, 95% CI 2.68 to 3.44).

### UV exposure and sun-protective behaviours by sex

We identified significant differences in sun protection behaviours and UV exposure between men and women (figure 1C,D). Women were significantly less likely to spend ≥2 hours in the sun compared with men (OR 0.69, 95% CI 0.62 to 0.76). Women had 185% higher odds of using sunscreen on their body (OR 2.85, 95% CI 2.68 to 3.03) and 322% higher odds of using it on their face (OR 4.22, 95% CI 3.96 to 4.49) compared with men, although they were less likely to use SPF ≥30 for both the body (OR 0.87, 95% CI 0.81 to 0.94) and face (OR 0.72, 95% CI 0.67 to 0.78). Women were also significantly less likely to wear sun-protective clothing, including long pants or skirts (OR 0.56, 95% CI 0.53 to 0.60), hats (OR 0.48, 95% CI 0.45 to 0.52) and sunglasses (OR 0.48, 95% CI 0.45 to 0.52). Women had a lower likelihood of sunburn (OR 0.85, 95% CI 0.80 to 0.90), but were far more likely to use tanning beds (OR 3.00, 95% CI 2.75 to 3.28).

### UV exposure and sun-protective behaviours by income quintile

Our findings indicate significant variations across income quintiles (figure 1E,F). Time spent in the sun increased proportionally with income, with only 34.7% of individuals in the lowest quintile (quintile 1) spending ≥2 hours in the sun, compared with 56.8% of individuals in the highest quintile (quintile 5). Individuals in the highest income quintiles had 78% higher odds of using any sunscreen on their body (OR 1.78, 95% CI 1.55 to 2.04) and 145% higher odds of using it on their face (OR 2.45, 95% CI 2.10 to 2.86), compared with the lowest quintile. Notably, differences in the use of sunscreen with SPF ≥30 on the body and face did not reach statistical significance. Similarly, while tanning bed use increased with income, differences across quintiles did not reach statistical significance.

The use of long pants and skirts was inversely related to income, with the highest quintile showing decreased odds (OR 0.45, 95% CI 0.40 to 0.51) compared with the lowest quintile. Hat-wearing did not significantly vary across quintiles, while sunglasses usage increased with income. Sunburn incidence also increased with income; individuals in the highest quintile had significantly higher odds of sunburn in the previous 12 months (OR 1.66, 95% CI 1.46 to 1.87).

### UV exposure and sun-protective behaviours by educational attainment

**Figure 2:**
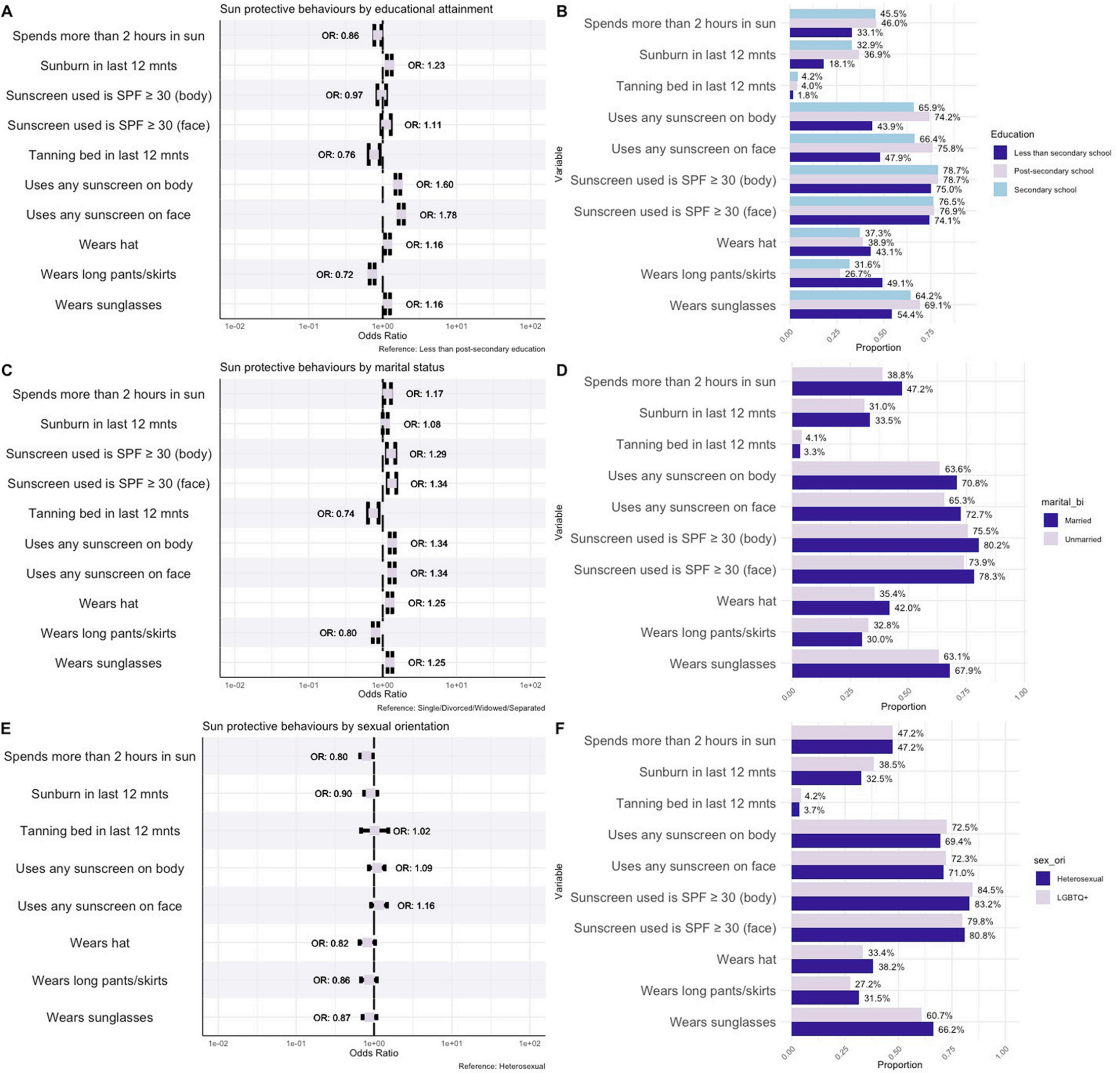
Sun exposure and protective behaviours by educational attainment, marital status and sexual orientation. Forest plot comparing sun exposure and protective behaviour outcome variables by educational attainment (**A**), marital status (**C**) and sexual orientation (**E**). Adjusted ORs controlling for the effect of age, sex and race, where applicable, are presented. Corresponding bar plots comparing the same sun exposure and protective behaviour variables by educational attainment (**B**), marital status (**D**) and sexual orientation (**F**). SPF, sun protection factor.

Differences in sun-protective behaviours between individuals with postsecondary education, compared with individuals with less than postsecondary education, are detailed in figure 2A,B. While individuals with postsecondary education were less likely to spend ≥2 hours in the sun compared with less educated individuals (OR 0.86, 95% CI 0.76 to 0.96), the prevalence of sunburn and tanning bed use did not significantly differ across groups, with ORs of 1.23 (95% CI 1.13 to 1.34) and 0.76 (95% CI 0.69 to 0.84), respectively.

Individuals with postsecondary education were more likely to use any sunscreen on their body (OR 1.60, 95% CI 1.49 to 1.72) and face (OR 1.78, 95% CI 1.65 to 1.93). The likelihood of wearing long pants or skirts was lower among educated individuals (OR 0.72, 95% CI 0.67 to 0.77), whereas the use of hats and sunglasses was slightly higher.

### UV exposure and sun-protective behaviours by marital status

Married individuals reported higher rates of sun exposure. The odds of having experienced sunburn in the last 12 months were slightly higher among married individuals (OR 1.08, 95% CI 1.00 to 1.17). Additionally, married individuals were more likely to spend ≥2 hours in the sun (OR 1.17, 95% CI 1.05 to 1.30), although single, widowed, divorced and separated individuals had higher odds of using tanning beds in the past 12 months (OR 0.74, 95% CI 0.63 to 0.88).

In our analysis, married individuals demonstrated higher odds of engaging in several protective behaviours compared with those who were single, widowed, divorced or separated (figure 2C,D). Married individuals had 34% higher odds of using sunscreen on the body and face (OR 1.34, 95% CI 1.24 to 1.45). Similarly, the odds of using sunscreen with SPF ≥30 on the body were increased (OR 1.29, 95% CI 1.13 to 1.47), as well as on the face (OR 1.34, 95% CI 1.18 to 1.52). Moreover, married individuals had greater odds of wearing hats (OR 1.25, 95% CI 1.16 to 1.34) and sunglasses (OR 1.25, 95% CI 1.16 to 1.34) compared with their single, widowed, divorced or separated counterparts, although they were less likely to wear long pants or skirts (OR 0.80, 95% CI 0.73 to 0.87).

### UV exposure and sun-protective behaviours by sexual orientation

The study also explored sun protection behaviours and UV exposure among lesbian, gay, bisexual, transgender and queer (or ‘questioning’) (LGBTQ+) individuals (figure 2E,F). LGBTQ+ individuals were less likely to spend ≥2 hours in the sun compared with heterosexual individuals (OR 0.80, 95% CI 0.70 to 0.91). UV exposure outcomes such as sunburn, tanning beds and sunscreen use did not significantly differ. The likelihood of wearing long pants or skirts was lower (OR 0.86, 95% CI 0.76 to 0.98) among LGBTQ+ individuals, as was wearing a hat (OR 0.82, 95% CI 0.72 to 0.94) and sunglasses (OR 0.87, 95% CI 0.76 to 1.00).

### UV exposure and sun-protective behaviours by immigration status

**Figure 3:**
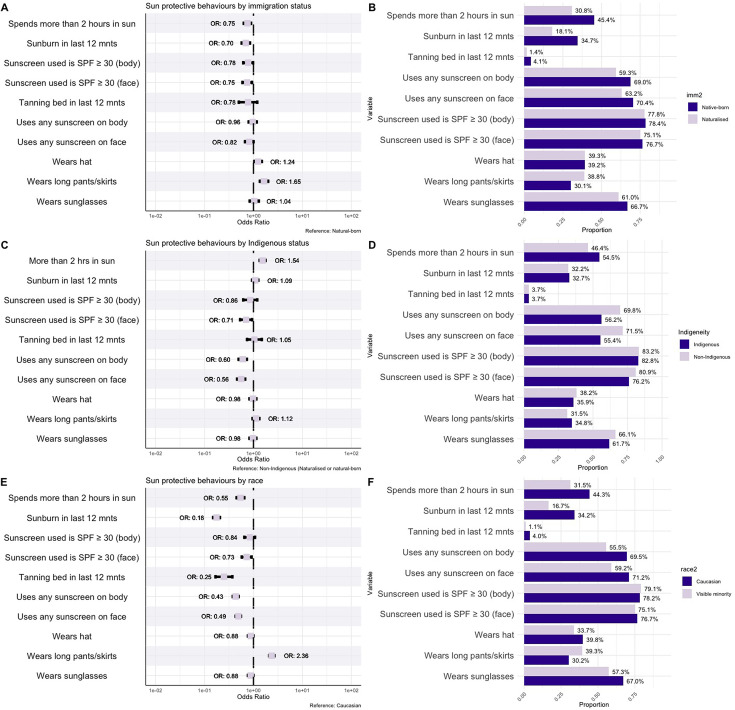
Sun exposure and sun-protective behaviours by immigration status, indigeneity and race. Forest plot comparing sun exposure and protective behaviour outcome variables by immigration status (**A**), indigeneity (**C**) and race (**E**). Adjusted ORs controlling for the effect of age, sex and race, where applicable, are presented. Corresponding bar plots comparing the same sun exposure and protective behaviour variables by immigration (**B**), indigeneity (**D**) and race (**F**). SPF, sun protection factor.

We identified differences in sun-protective behaviours among naturalised Canadians (immigrants) compared with the native-born population (figure 3A,B). Naturalised Canadians were less likely to spend time in the sun (OR 0.75, 95% CI 0.64 to 0.93) and less likely to have a sunburn in the previous 12 months (OR 0.70, 95% CI 0.64 to 0.77). Regarding sunscreen use, naturalised Canadians were less likely to use any sunscreen on their body (OR 0.96, 95% CI 0.88 to 1.04) and face (OR 0.82, 95% CI 0.75 to 0.90) compared with native-born citizens. The use of SPF ≥30 sunscreen was also less common among naturalised citizens for both the body (OR 0.78, 95% CI 0.71 to 0.86) and face (OR 0.75, 95% CI 0.68 to 0.83). However, naturalised Canadians were significantly more likely to wear long pants or skirts (OR 1.65, 95% CI 1.51 to 1.80) and hats (OR 1.24, 95% CI 1.05 to 1.48). While naturalised Canadians were less likely to use a tanning bed/booth, the difference was not statistically significant (OR 0.78, 95% CI 0.50 to 1.20).

### UV exposure and sun-protective behaviours by indigeneity

Important differences were noted when comparing sun-protective behaviours among Indigenous and non-Indigenous Canadians (figure 3C,D). Indigenous Canadians had 54% higher odds of spending ≥2 hours in the sun (OR 1.54, 95% CI 1.34 to 1.76), although there were no significant differences in sunburn (OR 0.96, 95% CI 0.83 to 1.12) or tanning bed use (OR 0.94, 95% CI 0.81 to 1.08).

Indigenous individuals were significantly less likely to use sunscreen on their body (OR 0.60, 95% CI 0.53 to 0.68) and face (OR 0.56, 95% CI 0.49 to 0.65). Results for the use of SPF ≥30 sunscreen followed a similar trend, with Indigenous individuals having lower odds of SPF ≥30 use on both the body (OR 0.86, 95% CI 0.76 to 0.98) and face (OR 0.71, 95% CI 0.61 to 0.82). There were no statistically significant differences between Indigenous and non-Indigenous Canadians with regard to long-sleeve or long skirt, sunglasses or hat use.

### UV exposure and sun-protective behaviours by race

Differences in sun-protective practices among visible minorities and white/Caucasian Canadians are presented in figure 3E,F. The study found significant disparities in sun protection behaviours and UV exposure among visible minorities. White Canadians spent more time in the sun compared with visible minorities, with 47.5% of white Canadians reporting ≥2 hours in the sun compared with 31.7% among visible minorities. As expected, visible minorities were significantly less likely to experience sunburn (OR 0.18, 95% CI 0.16 to 0.21) and use tanning beds (OR 0.25, 95% CI 0.22 to 0.28).

Individuals identified as visible minorities were significantly less likely to use sunscreen on their body (OR 0.43, 95% CI 0.40 to 0.47) and face (OR 0.49, 95% CI 0.45 to 0.54). Similarly, they had lower odds of SPF ≥30 sunscreen use for both the body (OR 0.84, 95% CI 0.77 to 0.91) and face (OR 0.73, 95% CI 0.67 to 0.79). Conversely, visible minorities had 136% higher odds of wearing long pants or skirts (OR 2.36, 95% CI 2.15 to 2.58) compared with white Canadians. Hat and sunglasses usage did not vary significantly.

### Trends in UV exposure and sun-protective behaviours

**Figure 4:**
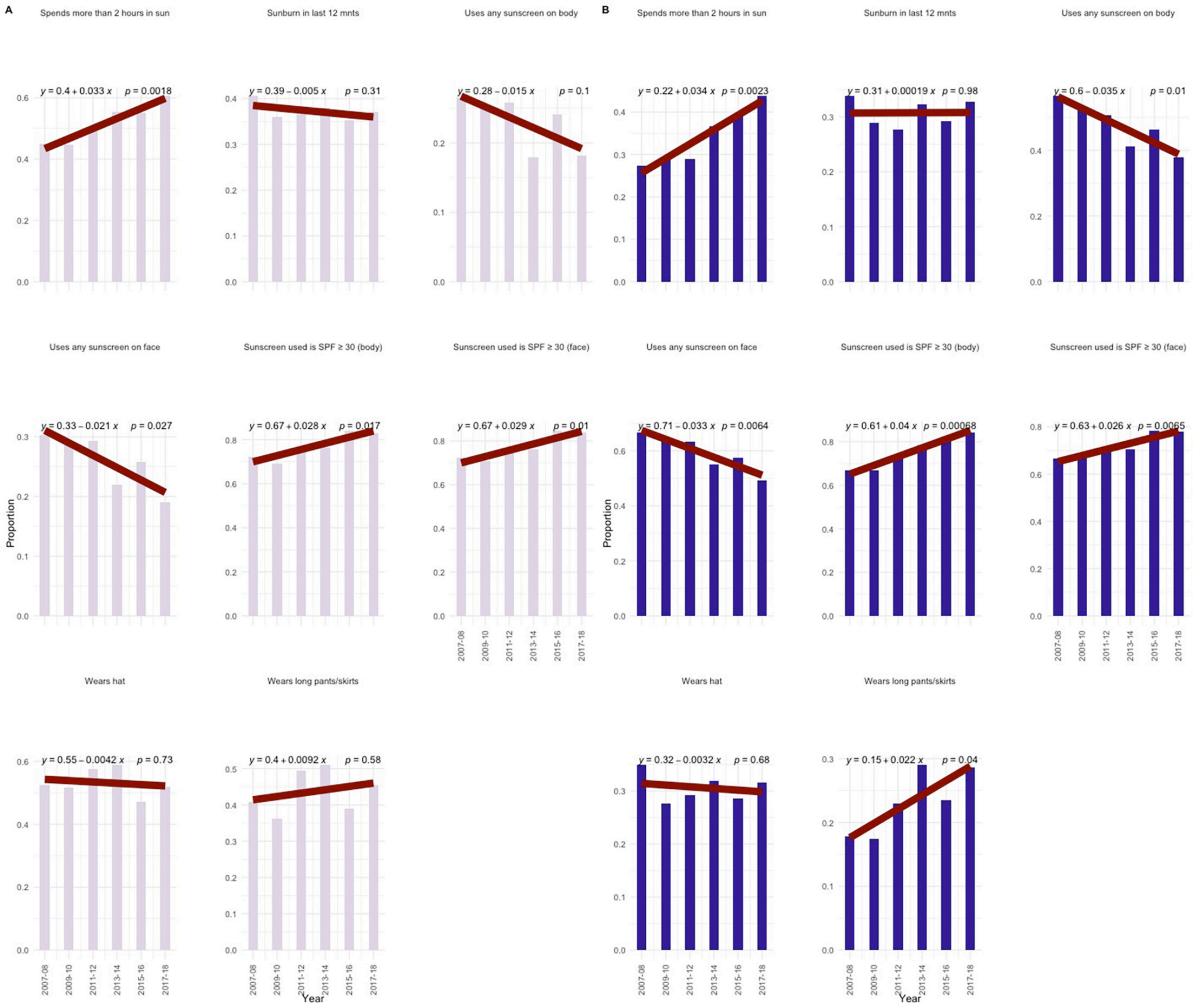
Trends in sun exposure and sun-protective behaviours for men and women, 2007–2018. Bar chart comparing sun exposure and protective behaviour outcome variables over time for men (**A**) and women (**B**). Sunscreen use variables are limited to individuals who report spending at least 30 min in the sun. SPF, sun protection factor.

Finally, our study explored temporal trends in UV exposure and sun-protective behaviours among Canadian men and women (figure 4A,B, respectively). We found a significant increasing trend among men and women spending ≥2 hours in the sun over time (p=0.0018 and p=0.0023, respectively). We noted a slight but insignificant decreasing trend in sunburn among men (p=0.31) and no detectable trend among women (p=0.98). We found that there was a decreasing trend in sunscreen use on the body for men (p=0.1) and for women (p=0.01), as well as on the face (p=0.027 and p=0.0064 for men and women, respectively). There was an increasing trend in the use of SPF ≥30 for both men and women on the body (p=0.017 for men and p=0.00068 for women) and on the face (p=0.01 for men and p=0.0065 for women). Finally, while there were no significant trends in hat wearing for men or women, the use of long pants/skirts increased over time among women (p=0.04).

## Discussion

The current study described alarming trends in UV exposure and sun-protective behaviours across major demographic and socioeconomic divisions in Canadian society. We found that 75.5% of Canadians spent at least 30 min in the sun between 10:00 and 16:00 hours on days off in the summer, with 45.7% spending ≥2 hours. One-third (33.3%) of Canadians reported having had a sunburn in the past 12 months, and most reported irregular or never use of sunscreen on their body (64.3%) and face (58.1%). Importantly, temporal trends showed an increasing prevalence of spending 2 hours or more in the sun and a decreasing trend in using sunscreen on the body and face for both men and women.

This study highlights significant differences in UV exposure and sun-protective practices by several independent demographic variables. Our study reveals age to be an important predictor of UV exposure after controlling for the effects of race and sex, with the youngest age group (18–29 years), unfortunately, having 54% higher odds of spending more time in the sun, 90% higher odds of sunburn and 104% higher odds of tanning bed or booth use, compared with the oldest age group (≥70 years). In addition, young people were far less likely to use physical protection (hats and long pants/skirts). These data are corroborated by survey data in the USA, which has also shown young people aged 18–24 years to be less likely to protect themselves from the sun.^24^ Some literature suggests that sun exposure and tanning behaviour among youth is related more to a sense of invulnerability to the risks of UV exposure^25^ and the aesthetic appeal of looking tan,^26 27^ rather than to a lack of knowledge. We theorise that cost and lack of accessibility to sun protection products may also drive lower sun protection habits in these individuals.^20^ Future studies should aim to understand young adults’ attitudes towards sun exposure risks and the need for sun protection.

Income quintile also appears to be a significant predictor of UV exposure and sun-protective practices after controlling for the effects of sex and race.^11 18 19 28 29^ There were increasing trends in time spent in the sun, odds of sunburn and odds of tanning bed/booth use with increasing income quintile. The highest income quintile had 78% higher odds of using sunscreen on the body, and 145% higher odds of using sunscreen on the face, compared with the lowest income earners. These results may indicate financial barriers related to buying sunscreen, given 8 ounces of commercial sunscreen can cost between US$21 and US$58.^30^ In Canada, there are no sales tax exemptions related to sunscreen products.^31^ These findings are important given evidence suggesting that higher socioeconomic status (SES) is related to increased incidence of CM, but populations of lower SES have an increased risk of advanced melanoma diagnosis.^32^ Increasing accessibility and education around sun protection may thus potentially avoid some of the most serious cases of CM. Indeed, campaigns like Australia’s SunSmart health promotion campaign^33 34^ and the implementation of tax exemptions for sun safety products^31^ have demonstrated that targeted public health and policy interventions can effectively reduce sunburn prevalence and change sun-related behaviours and attitudes at a population level.

Immigrants, Indigenous individuals and visible minorities were less likely to use sunscreen on their face and body compared with their counterparts, although they were more likely to wear long pants or skirts as protective barriers.^35^ Given that visible minorities are often thought to experience fewer harmful effects from sunlight, the need for sun protection in this group is less studied and may be underestimated.^36^ Our study reveals significant disparities in the use of sunscreen among these groups, and future research is warranted to understand perceptions of skin cancer risk among these populations, especially given recent evidence of racial and ethnic disparities in melanoma survival.^37^

The large sample size of the CCHS and its more recent inclusion of the sun protection modules have offered a unique opportunity to understand Canadian attitudes and behaviours towards sun exposure and protection, highlighting numerous high-risk behaviours and worrisome trends.

Nevertheless, there are important limitations associated with this study. The CCHS sampling method makes it broadly representative of the provinces included in this study, although it is likely not generalisable to all Canadians. The CCHS relies on self-reported data, which makes the study prone to recall and social desirability biases. The data provide a cross-sectional evaluation of dependent variables and cannot be used to understand chronic UV exposure or longitudinal changes in sun exposure behaviours over time. Variables were grouped dichotomously to maintain anonymity and enhance the interpretability of results, particularly given the large dataset. Nevertheless, dichotomous variables often oversimplify complex social and demographic identities, potentially obscuring within-group heterogeneity and limiting the ability to capture nuanced differences in behaviours and outcomes.

## Conclusion

This comprehensive analysis of the CCHS highlights significant demographic and socio-economic disparities in sun protection behaviours and UV exposure across Canada. Despite increased awareness of melanoma risks, trends indicate a troubling rise in sun exposure over the years, coupled with inconsistent sunscreen use. These findings underscore the urgent need for targeted public health interventions and policies to promote effective sun safety behaviours, particularly among high-risk populations.

## Supplementary material

10.1136/bmjph-2024-001983 online supplemental file 1

## Data Availability

Data are available in a public, open access repository.
