# Supplementary material for: Evaluating UV exposure and skin cancer prevention behaviours in Canada: a national population-based cross-sectional study
Source: BMJ Public Health. 2025 Apr 23;3(1):e001983. doi: 10.1136/bmjph-2024-001983 (PMC12020754; doi:10.1136/bmjph-2024-001983)
Supplement: online supplemental file 1 [file bmjph-3-1-s001.docx]

| **Supplementary Table 1.** Summary Characteristics of Canadian Community Health Survey Respondents (2007-2018) | | |
| --- | --- | --- |
|  | Unweighted sample  N_unweighted_ = 106,060 | Weighted sample  N_weighted_ = 28,845,617 |
| Characteristics | n (%) | n (%) |
| **Sex** |  |  |
| Male | 48062 (45.3%) | 14144211 (49.0%) |
| Female | 57998 (54.7%) | 14701407 (51.0%) |
| **Age** |  |  |
| 18-29 | 15037 (14.2%) | 5593473 (19.4%) |
| 30-39 | 15083 (14.2%) | 4822726 (16.7%) |
| 40-49 | 14587 (13.8%) | 5054746 (17.5%) |
| 50-59 | 19589 (18.5%) | 5374816 (18.6%) |
| 60-69 | 20652 (19.5%) | 4379650 (15.2%) |
| 70+ | 21112 (19.9%) | 3620206 (12.6%) |
| **Race** |  |  |
| White | 90900 (90.8%) | 22022127 (81.3%) |
| Visible Minority | 9191 (9.2%) | 5056154 (18.7%) |
| **Educational Attainment** |  |  |
| Less than Secondary School Graduation | 20233 (19.4%) | 4078762 (14.4%) |
| Secondary School Graduation | 62589 (60.1%) | 18423443 (65.1%) |
| Post-Secondary School | 21318 (20.5%) | 5786844 (20.5%) |
| **Household Income Quintile** |  |  |
| 1 | 21787 (21.3%) | 5464101 (19.7%) |
| 2 | 20844 (20.4%) | 5553192 (20.0%) |
| 3 | 19874 (19.5%) | 5527428 (19.9%) |
| 4 | 19562 (19.2%) | 5617557 (20.2%) |
| 5 | 20000 (19.6%) | 5636579 (20.3%) |
| **Province** |  |  |
| Manitoba (MB) | 10013 (10.2%) | 1921620 (6.9%) |
| New Brunswick (NB) | 4454 (4.5%) | 596314 (2.1%) |
| Ontario (ON) | 30308 (31.0%) | 10983554 (39.4%) |
| Quebec (QC) | 42084 (43.0%) | 12767500 (45.8%) |
| Saskatchewan (SK) | 11032 (11.3%) | 1627540 (5.8%) |
| **Immigration status** |  |  |
| Native-born | 90108 (87.4%) | 21417511 (77.1%) |
| Naturalized | 12937 (12.6%) | 6360177 (22.9%) |
| **Indigeneity** |  |  |
| General Population/Non-Indigenous | 62913 (95.4%) | 19685206 (96.8%) |
| Indigenous | 3034 (4.6%) | 658923 (3.2%) |
| **Time spent in sun** |  |  |
| 1. 0 to < 30 minutes | 27398 (27.8%) | 7061450 (26.3%) |
| 2. 30 minutes to < 2 hours | 28362 (28.8%) | 7980964 (29.7%) |
| 3. 2 to < 4 hours | 27838 (28.3%) | 7761770 (28.9%) |
| 4. 4 to 6 hours | 14883 (15.1%) | 4088333 (15.2%) |
| **Sunburn in the last twelve months** |  |  |
| No | 70062 (67.6%) | 18556589 (66.1%) |
| Yes | 33524 (32.4%) | 9501082 (33.9%) |
| **Tanning bed or booth in the last twelve months** |  |  |
| No | 61902 (96.3%) | 18998933 (96.4%) |
| Yes | 2356 (3.7%) | 713526 (3.6%) |
| **Uses sunscreen on body** |  |  |
| Sometimes/rarely/never | 48063 (64.3%) | 13131291 (63.6%) |
| Always/often | 26714 (35.7%) | 7525194 (36.4%) |
| **Uses sunscreen on face** |  |  |
| Sometimes/rarely/never | 42938 (57.4%) | 11819990 (57.2%) |
| Always/often | 31909 (42.6%) | 8847497 (42.8%) |
| **Sun Protection Factor (SPF) of sunscreen used (body)** |  |  |
| ≤ SPF 29 | 8236 (21.6%) | 2278492 (21.0%) |
| ≥ SPF 30 | 29824 (78.4%) | 8578585 (79.0%) |
| **Sun Protection Factor (SPF) of sunscreen used (face)** |  |  |
| ≤ SPF 29 | 9743 (23.4%) | 2632795 (22.4%) |
| ≥ SPF 30 | 31810 (76.6%) | 9123560 (77.6%) |
| **Wears hat** |  |  |
| Sometimes/rarely/never | 45493 (60.8%) | 13280752 (64.3%) |
| Always/often | 29334 (39.2%) | 7381536 (35.7%) |
| **Wears long pants/skirt** |  |  |
| Sometimes/rarely/never | 51453 (68.8%) | 14938001 (72.4%) |
| Always/often | 23291 (31.2%) | 5704894 (27.6%) |
| **Wears sunglasses** |  |  |
| Sometimes/rarely/never | 16242 (34.1%) | 5184069 (35.3%) |
| Always/often | 31405 (65.9%) | 9485296 (64.7%) |
|  | | |
